# Apoptosis by [Pt(*O*,*O′*-acac)(γ-acac)(DMS)] requires PKC-δ mediated p53 activation in malignant pleural mesothelioma

**DOI:** 10.1371/journal.pone.0181114

**Published:** 2017-07-12

**Authors:** Antonella Muscella, Carla Vetrugno, Luca Giulio Cossa, Giovanna Antonaci, Amilcare Barca, Sandra Angelica De Pascali, Francesco Paolo Fanizzi, Santo Marsigliante

**Affiliations:** 1 Laboratory of Cell Pathology, Department of Biological and Environmental Sciences and Technologies (Di.S.Te.B.A.), University of Salento, Lecce, Italy; 2 Laboratory of Cell Physiology Di.S.Te.B.A., University of Salento, Lecce, Italy; 3 Laboratory of Physiology Di.S.Te.B.A., University of Salento, Lecce, Italy; 4 Laboratory of Inorganic Chemistry, Di.S.Te.B.A., University of Salento, Lecce, Italy; Institute of Biochemistry and Biotechnology, TAIWAN

## Abstract

Mesothelioma cancer cells have epithelioid or sarcomatoid morphology. The worst prognosis is associated with sarcomatoid phenotype and resistance to therapy is affected by cells heterogeneity. We recently showed that in ZL55 mesothelioma cell line of epithelioid origin [Pt(*O*,*O′*-acac)(γ-acac)(DMS)] (Ptac2S) has an antiproliferative effect *in vitro* and *in vivo*. Aim of this work was to extend the study on the effects of Ptac2S on ZL34 cell line, representative of sarcomatoid mesothelioma. ZL34 cells were used to assay the antitumor activity of Ptac2S in a mouse xenograft model *in vivo*. Then, both ZL34 and ZL55 cells were used in order to assess the involvement of p53 protein in (a) the processes underlying the sensitivity to chemotherapy and (b) the activation of various transduction proteins involved in apoptosis/survival processes. Ptac2S increases ZL34 cell death *in vivo* compared with cisplatin and, *in vitro*, Ptac2S was more efficacious than cisplatin in inducing apoptosis. In Ptac2S-treated ZL34 and ZL55 cells, p53 regulated gene products of apoptotic BAX and anti-apoptotic Bcl-2 proteins via transcriptional activation. Ptac2S activated PKC-δ and PKC-ε; their inhibition by PKC–siRNA decreased the apoptotic death of cells. PKC-δ was responsible for JNK1/2 activation that has a role in p53 activation. In addition, PKC-ε activation provoked phosphorylation of p38MAPK, concurring to apoptosis. In ZL34 cells, Ptac2S also activated PKC-α thus provoking ERK1/2 activation; inhibition of PKC-α, or ERK1/2, increased Ptac2S cytotoxicity. Results confirm that Ptac2S is a promising therapeutic agent for malignant mesothelioma, giving a substantial starting point for its further validation.

## Introduction

The incidence of malignant pleural mesothelioma (MPM) is growing due to wide asbestos usage in various developing countries [1]. The most efficacious MPM treatment able to lengthen sufferers’ life is the combination of pemetrexed or raltitrexed, multi-folate inhibitors and cisplatin; nevertheless, the median survival is 12 months, with response rates of about 40% [2, 3]. Biologic agents targeting oncogenetic pathways, such as histone deacetylases, phosphatidylinositol 3-kinase /mammalian target of rapamycin, nuclear factor kB and neoangiogenesis have also been tested [4]. However, none of these treatments showed to impact significantly on this neoplasm; thus, there is an urgent need for new drugs. Histologically, MPM can be classified in the following three subtypes: epithelioid (50%), sarcomatoid (16%), and mixed type or biphasic (34%). Sarcomatoid mesotheliomas are characterized by aggressive biological behaviour, resistance to systemic treatments, more frequent distant spread and poor prognosis. Great care has been given on designing new platinum-based compounds having fewer toxicity and more favourable therapeutic indices than *cisplatin*. In regard to this, it was synthesized the Pt(II)-derived drug [Pt(*O*,*O'*-acac)(γ-acac)(DMS)] (Ptac2S) having non-genomic targets [5]. Ptac2S achieved increasing heed as potential anticancer drug as its high and selective cancer cell cytotoxicity observed in immortalized cell lines and in breast cancer cells in primary culture [6–10] and *in vivo* [11–13]. Notably, in a preclinical model made of hypodermic injection of breast cancer cells, Ptac2S shows up for an anticancer activity higher than cisplatin; in Wistar rats it as well showed increased pharmacokinetics, bio distribution and tolerability in comparison to cisplatin. Pharmacokinetics studies with Ptac2S uncovered lengthened systemic blood persistence of Pt and diminished nephrotoxicity and hepatotoxicity. In principle, this Pt-compound would yield a wider use, since Ptac2S also exerts specific antimetastatic responses in vitro [13–14]. As said, it seems notable to understand whether Ptac2S has also cytotoxic effects on MPM. Previously, we used the epithelioid ZL55 cells and showed that cisplatin provoked apoptosis together with the activation of PKC-α and ERK1/2 pro-survival pathways by the synthesis of ROS [15]. In the same ZL55 cells we also tested the effects of Ptac2S and observed a greater cytotoxicity than cisplatin. Ptac2S was able to activate different transduction pathways with strong pro-apoptotic activity (p38 and PKC-δ), while the PKC-α pro-survival pathway activated by cisplatin was not observed. Therefore, the higher cytotoxicity of Ptac2S in these cells may be due to the fact that it does not activate PKC-α [12]. In the current investigation, we assess the cytotoxicity of Ptac2S also on mesothelioma cells of sarcomatoid origin that are generally more aggressive and less susceptible to chemotherapy. Therefore, this study was conducted using the ZL34 cells both *in vitro* and *in vivo* with the technique of the xenograft on nude mice. Furthermore, we also looked for the differences between responses to Ptac2S and cisplatin and the molecular mechanisms that determine the ZL34 cell death/survival fate.

## Materials and methods

### Cell culture

The human mesothelioma cell lines ZL34 and ZL55 [15] were grown in RPMI 1640 medium (Sigma, St. Louis, MO, USA) supplemented with 10% fetal bovine serum (FBS), penicillin (100 U/ml) and streptomycin (100 mg/ml). The cells were maintained at 37°C in the presence of 5% CO_2_ in air. Cells were grown to 70–80% confluence and then treated with Pt-compounds at various concentrations and for different incubation periods.

### *In vivo* xenograft experiments

Athymic nude mice (6 wks. old, female, 20 to 30 g body weight) were purchased from Harlan Laboratories (San Pietro al Natisone UD, Italy) and maintained under pathogen-free conditions. They were given free access to standard food and water, with a 12 h light-dark cycle at a temperature of 22+/−2°C. Approximately 6 x 10^6^ ZL34 cells (8 mice) were injected subcutaneously into the flank. Animals were monitored daily for general health and body weights were measured twice weekly. Tumour size was measured with slide callipers and volumes were calculated as (LxW^2^)/2, where L and W are the major and minor diameters, respectively. Once tumour volumes reached ~50 mm^3^, mice were randomly divided into three groups and treated by a single intravenous of saline as a control, or 10 mg/kg of Ptac2S or 10 mg/kg cisplatin. The mice were sacrificed after 35 days of treatment and the tumours were excised. As described previously [11], all animals received care in compliance with the Principles of Laboratory Animal Care formulated by the National Society for Medical Research and the Guide for the Care and Use of Laboratory Animals prepared by the Institute of Laboratory Animal Resources, published by the National Institutes of Health (NIH Publication No. 86–23, revised 1985), as well as in accordance with the Italian laws on animal experimentation (art. 4 and 5 of D.L. 116/92). Ethical Committee on Animal Research (Ministero della Salute D.M. 109/2014-B) approved the protocols. All efforts were made to minimize suffering to animals; thus, the experimental procedures used in the work described in this article were in compliance with the guidelines for reporting experiments involving animals [16].

### Cytotoxicity assay

We evaluated the IC_50_ in ZL34 cells with SRB and MTT assays. The SRB (sulforhodamine B) assay and the conversion of MTT (3-(4,5-dimethylthiazol-2-yl)-2,5-diphenol tetrazolium bromide) by mesothelioma cells were used as indicator of cell number as described previously [7]. Viable cells were also counted by the trypan blue exclusion assay and light microscopy. The data presented are means ± standard deviation (S.D.) from eight replicate wells per microtitre plate.

### Clonogenic survival assay

ZL34 cells were seeded in 100 mm Petri dishes at low density (~3X10^4^ per dish) and left to adhere for 24 h in a standard medium. Crescent concentrations of Ptac2S or cisplatin were added and clonogenic survival assay was performed as described previously [8].

### Preparation of subcellular fraction and western blots

Preparation of sub cellular fraction, western blotting analysis and immunodetection were performed as previously reported [17]. Western blotting and immunodetection analyses were performed as previously described [18].

### Reverse transcription and polymerase chain reaction (RT-PCR)

Total RNA was extracted from ZL34 and ZL55 cells using an SV Total RNA isolation kit and performed according to the manufacturer’s protocols (Promega, Madison, WI, USA) as previously described [8]. A melt curve analysis was performed following every run to ensure a single amplified product for every reaction. For each gene, relative expression was determined using the 2^-ΔΔCT^ methods and normalized to β-actin expression.

### Design and preparation of small interfering RNA (siRNA)

PKC-α, PKC-δ and PKC-ε siRNAs were prepared by an *in vitro* transcription method, according to the manufacturer’s protocol (Promega, Madison, WI, USA) as previously described [8].

### siRNA transfection

The cells (50–70% confluence) were transfected with siRNA duplexes using the protocol supplied with the CodeBreaker siRNA transfection reagent (Promega, Madison, WI, USA) as described previously [15]. Quantitative analysis of protein expression, as measured by intensity of immunoreactivity in siRNA-transfected cells, revealed a very high reduction in PKC-α, PKC-δ and PKC-ε expression.

### Statistical analysis

The experimenter measuring the tumours and the data analyst were unaware of the treatments given to the animals. Data, presented as means ± SD, were collected in blinded fashion and analysed using GRAPHPAD PRISM 5 software (GraphPad Software, La Jolla, CA, USA). Unpaired Student’s t-test or one-way ANOVA, and when this returned P < 0.05, post hoc analysis using Bonferroni test, were performed; we used the Bonferroni-Dunn post hoc test in the ANOVA after a significant omnibus F-test. P < 0.05 was accepted as a level of statistical significance.

#### Materials

Ptac2S was prepared as previously reported [5, 19]. Cisplatin was purchased from Sigma (Milan, Italy). RPMI 1640 medium, antibiotics, glutamine and foetal bovine serum were purchased from Celbio (Milan, Italy). Caspase -9 and -7, BAX, PARP-1, phospho p38MAPK, phospho JNK1/2 antibodies were obtained from Cell Signalling (Celbio, Milan, Italy). PKC isoforms and phospho ERK1/2 antibodies, goat anti-rabbit conjugated with peroxidase, as well as control antibodies were obtained from Santa Cruz Biotechnology (Santa Cruz, CA, USA). All others reagents were from Sigma (Milan, Italy).

## Results

### Anticancer activity of Ptac2S in a MPM preclinical model

**Fig 1:**
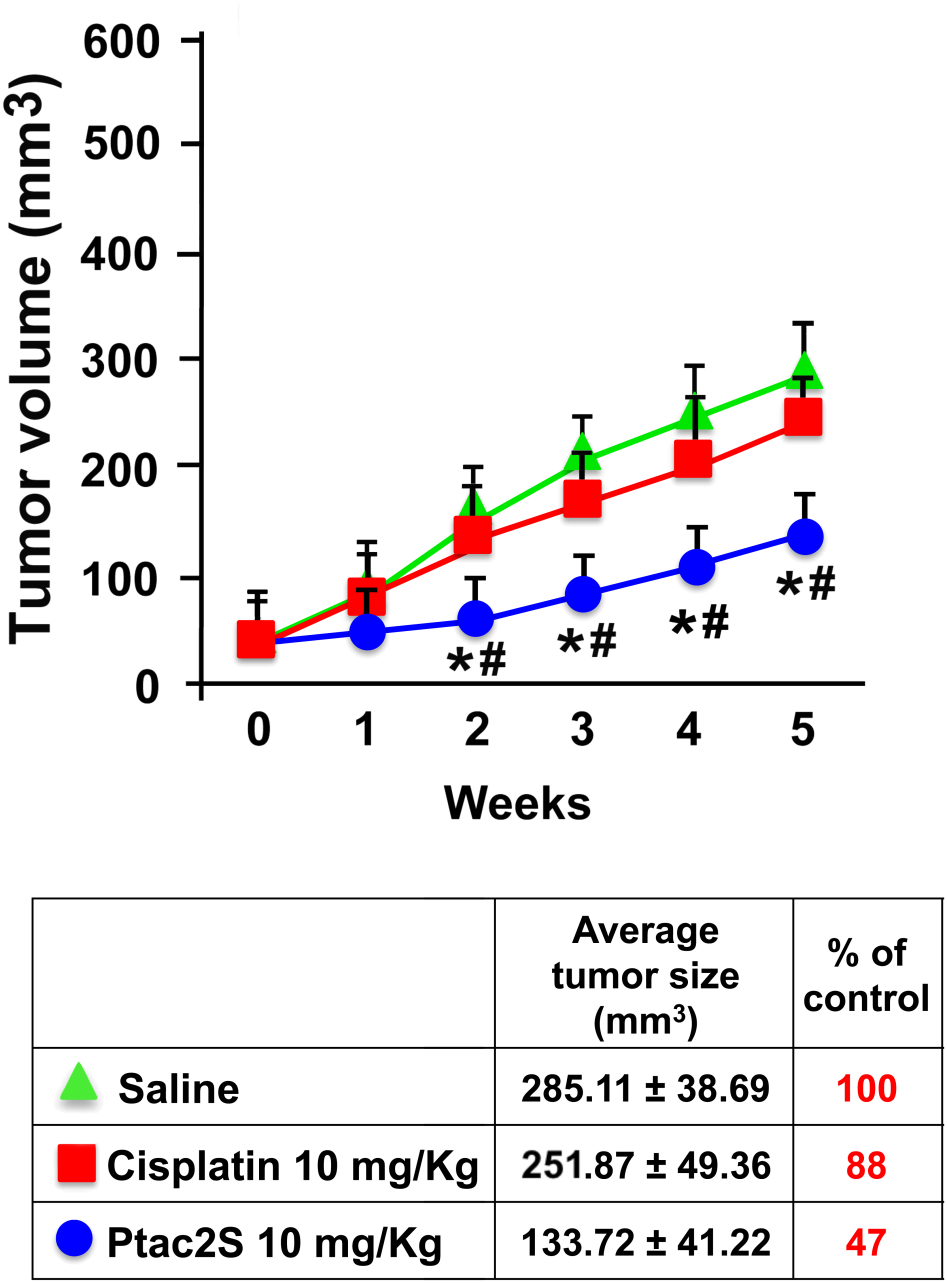
Growth inhibitory effect of Ptac2S and cisplatin in a xenograft model of mesothelioma. Balb/c nude mice carrying sarcomatoid or epithelioid mesothelioma developed by injection of ZL34 (around 50 mm^3^) received intravenous Ptac2S (10 mg/kg) or cisplatin (10 mg/kg). Tumour volume was measured every 3 days for a total of 35 days. Results are showed as mean ± S.D. (animals per group n = 8). *P < 0.05, significantly different from saline control; #P < 0.05, significantly different between Ptac2S and cisplatin. **(Table)** After killing, tumours were collected and measured.

ZL34 and ZL55 cell lines represent sarcomatoid and epithelioid MPM, respectively. The *in vivo* efficacy of Ptac2S in preclinical model of epithelioid MPM was already determined [12]. Here, sarcomatoid model was assessed by the hypodermic injection of ZL34 cells in the flank of BALB/c nude mice. When cancers reached the size of ~50 mm^3^, in order to reduce weight and tumour size odds, mice were randomized in three groups. 10 mg/kg of Ptac2S was found before to be effective without notable side effects in animal studies with xenografts of human breast cancerous cells [11]. Hence, after that an only intravenous of saline (control) or 10 mg/kg of Ptac2S or 10 mg/kg of cisplatin was dispensed, tumour volumes were evaluated by vernier calliper every 3 days for 5 weeks. The mean volumes of the tumours in each group were assessed, and we drew the related tumour growth curves. Ptac2S displayed higher anticancer activity than cisplatin toward ZL34 tumours examined, inducing up to 50% growth inhibition. In mice inoculated with ZL34, during 5 weeks mean tumour volume augmented from 46.6±6.78 to 285.11±38.69 mm^3^ for the saline group, to 251.87±49.36 mm^3^ for the cisplatin group (10mg/kg; p>0.05) and to 133.72±41.22 mm^3^ for the Ptac2S group (Fig 1). Mice displayed a significant decrease of tumour mass for each experimental time considered in the Ptac2S groups compared with both control and cisplatin-treated mice (p < 0.05). In addition, during observation time, no health problems were observed and the overall behaviour was similar to that of the control animals.

### Cytotoxicity of Ptac2S

**Fig 2:**
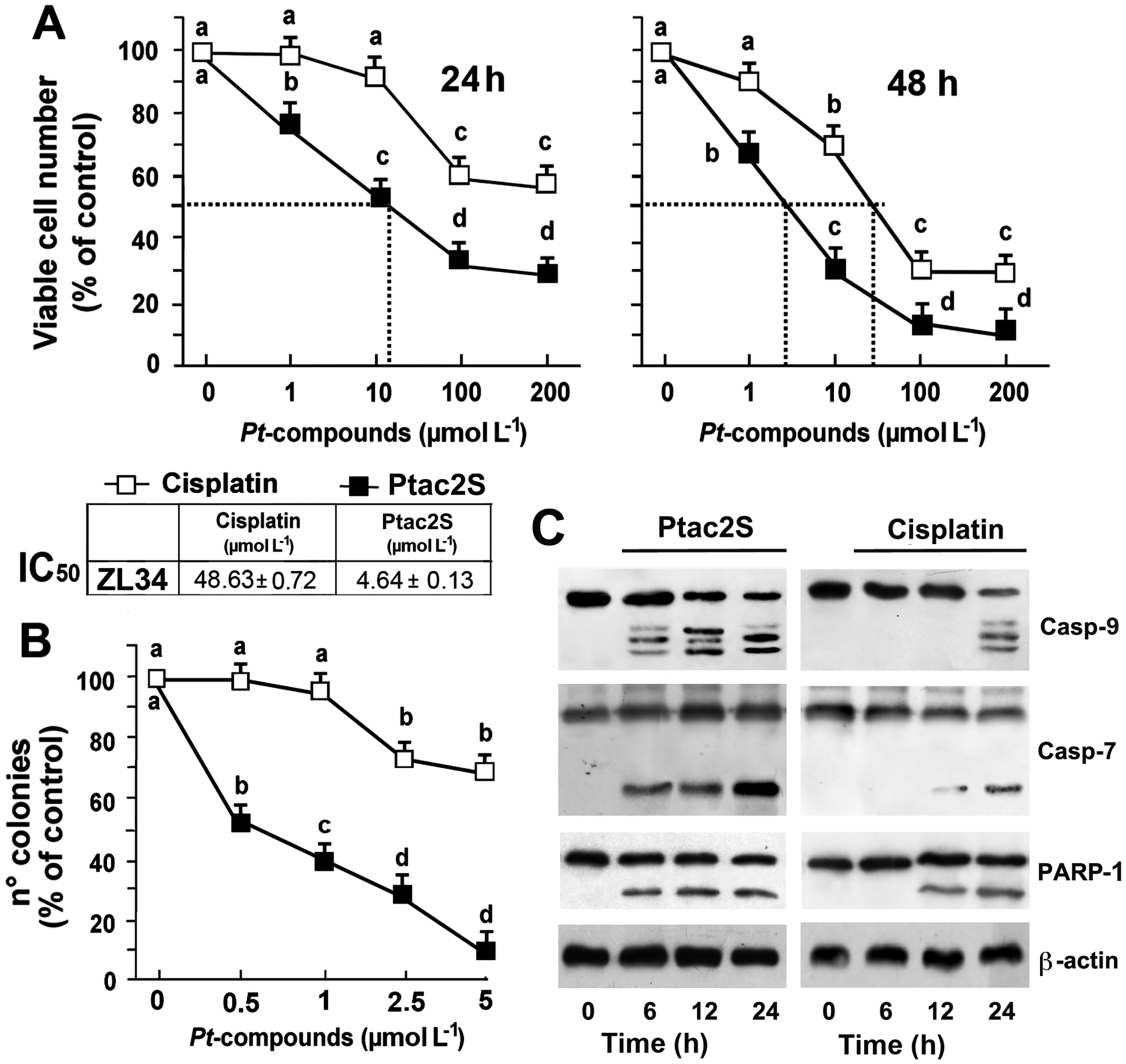
Sensitivity of MPM cells to Ptac2S and cisplatin. **(A)** ZL34 cells were treated or not with increasing concentrations of Ptac2S or cisplatin for 24 and 48 h, or continuously exposed to 50 μM cisplatin or 5 μM Ptac2S. Cell viability was obtained by SRB assay and data are means ± S.D. of 6 independent experiments with 8 replicates in each, and are presented as per cent of control. For both cisplatin and Ptac2S, P < 0.0001 by one-way ANOVA (n = 6); values with shared letters are not significantly different according to Bonferroni/Dunn post hoc tests. **(B)** Clonogenic survival assay in ZL34 cells treated with the indicated amounts of Ptac2S or cisplatin for 2 h, and after 15 days of growth; only colonies consisting of more than 50 cells were scored. The percentage of number colonies represents the means ± S.D. of six-independent experiments. For cisplatin and Ptac2S, P < 0.001 and P<0.0001 by one-way ANOVA (n = 6), respectively; values with shared letters are not significantly different according to Bonferroni/Dunn post hoc tests. **(C)** Cytosolic and nuclear proteins were obtained from ZL34 cells treated or not with 5 μM Ptac2S or 50 μM cisplatin. Samples were dissolved in SDS buffer and separated on SDS gel. Immunoblotting was performed using monoclonal antibodies specific to PARP (from nuclear fractions) and to caspases-9, and -7 (cytosolic fractions). Sequential incubation with anti-β-actin confirmed the equal protein loading. Figures are representative of 6 independent experiments. **Inset**: The IC_50_ values to cisplatin and Ptac2S calculated after 48 h.

*In vitro* cytotoxicity data were achieved by MTT and validated by SRB assays to exclude consequences of Ptac2S on enzymes of mitochondria. Furthermore, similar results are attained when cell number is defined through their counting (data not shown); thus, SRB assay was used for all the experiments shown herein. A dose-dependent decrement of cell survival was obtained when MPM cells were incubated with cisplatin or Ptac2S (from 1 μM to 200 μM, Fig 2A). In sarcomatoid cells cisplatin was significantly less cytotoxic than Ptac2S (IC_50_ 4.64±0.13 and 48.63±0.72 μM n = 6, for Ptac2S and cisplatin, respectively; p<0.001, see table in Fig 2). Clonogenic assay performed on ZL34 showed that Ptac2S was more cytotoxic than cisplatin (Fig 2B).

### Ptac2S causes caspases proteolysis

Fig 2C shows western blotting of caspase-9 and -7 activation and proteolysis of PARP in ZL34 and ZL55 cells. PARP was cleaved in cells treated with 5 μM Ptac2S or with 50 μM cisplatin; however, proteolysis was faster with Ptac2S. Subsequent incubation of blots with an antibody against β-actin validated that loaded protein amount was the same.

### Ptac2S induces p53 activation

**Fig 3:**
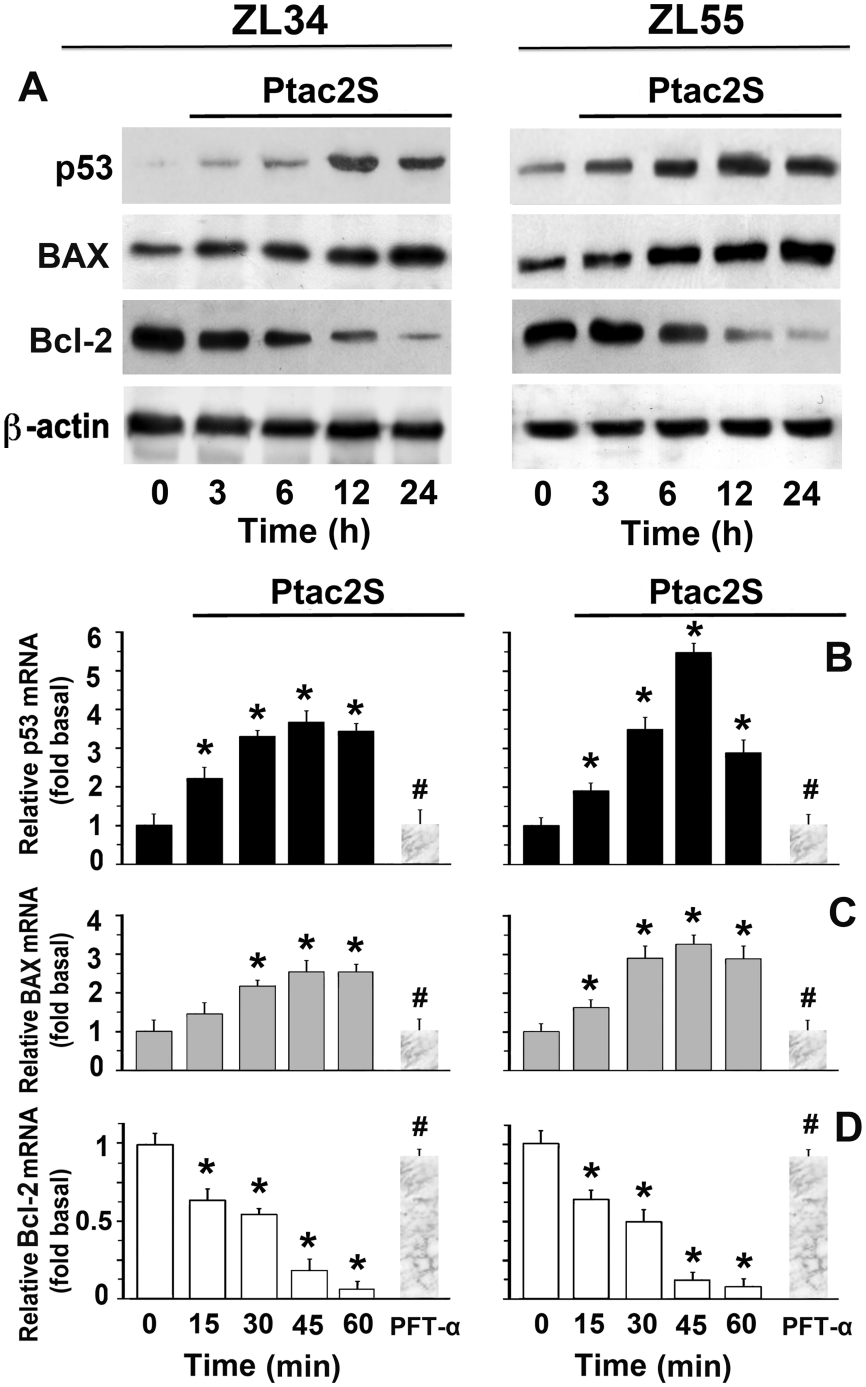
Ptac2S induces p53 in MPM cells. **(A)** Cytosolic proteins were obtained from ZL34 or ZL55 cells, treated or not with 5 μM Ptac2S. Immunoblotting was performed using monoclonal antibodies specific to p53, Bax and Bcl2. Sequential incubation with anti-β-actin confirmed the equal protein loading. These figures are representative of six independent experiments. **(B-D)** Cells, pre-treated or not with 30 μM PFT-α, were treated or not with 5 μM Ptac2S for different times and then RNA was extracted. RNA was reverse-transcribed and analysed by real-time PCR, with specific primers for p53 (**B**), BAX (**C**) and Bcl-2 (**D**) and for the housekeeping gene β-actin. mRNA levels were presented as fold change values relative to control. Data were expressed as the mean ± S.D. six different experiments. *P < 0.05, significantly different from saline control; #P < 0.05, significantly different between Ptac2S and PFT-α.

**Fig 4:**
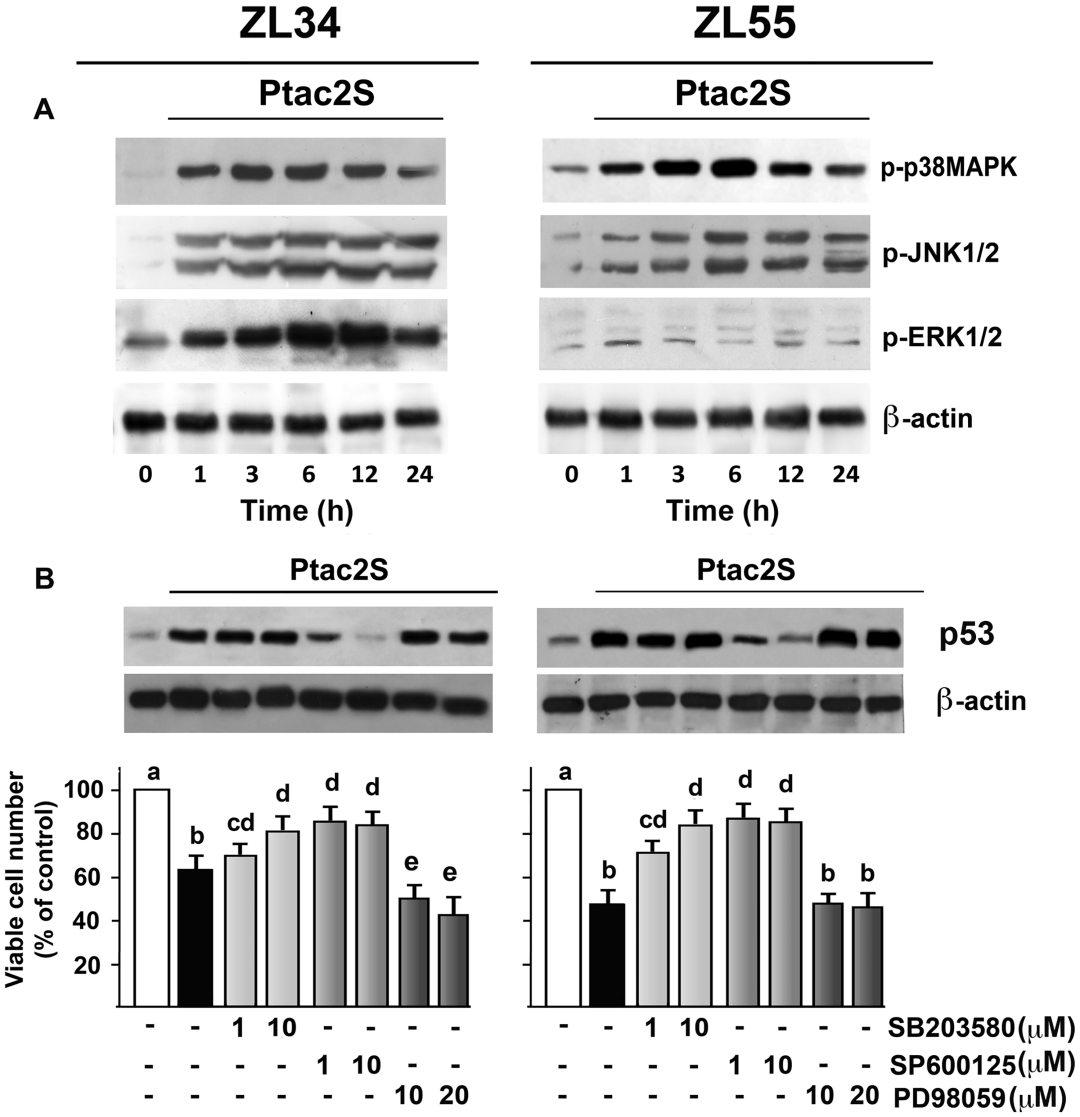
Ptac2S induces MAPKs phosphorylation in MPM cells. (**A**) ZL34 and ZL55 cells were treated or not with 5 μM Ptac2S for indicated time. Cell lysates were analysed by western blotting with anti-phosphorylated p38MAPK, JNK1/2 and ERK1/2 antibodies. (**B**) Cells, pre-treated or not with the p38MAPK inhibitor SB203580 (1 and 10 μM), the JNK inhibitor SP600125 (1 and 10 μM) or MEK inhibitor (10 and 20 μM), were then incubated with Ptac2S. Cell lysates were analysed by western blotting using monoclonal antibody specific to p53. Sequential incubation with anti-β-actin confirmed the equal protein loading. These figures are representative of six independent experiments. Viable cell number was determined 24 h later by SRB assay. The data are means ± S.D. of five different experiments run in eight replicates and are presented as percent of control. P < 0.0001 by one-way ANOVA (n = 5); values with shared letters are not significantly different according to Bonferroni/Dunn post hoc tests.

Since drugs may stabilize p53, that is not mutated in many MPM specimens [20], we assessed Ptac2S effects on p53 and its related genes Bcl-2 and BAX. Ptac2S treatment increased p53 protein levels (Fig 3A), increased BAX and decreased Bcl-2 proteins (Fig 3A) in both ZL34 and ZL55 cells. By RT-PCR we found that Ptac2S up-regulated p53 and BAX mRNA expression, and decreased Bcl-2 mRNA in a time-dependent way (ANOVA p < 0.01, Fig 3B, 3C and 3D). We next used an inhibitor of p53 transcriptional targets, PFT-α [21]. Fig 4 shows that 30 μM PFT-α inhibited p53 and BAX up-regulation and Bcl-2 down-regulation due to Ptac2S in both cell lines, indicating that apoptosis induced by Ptac2S was mediated by p53 and BAX.

### Ptac2S-induces MAPKs phosphorylation

We demonstrated previously that Ptac2S activates the MAPKs signalling pathways in several tumour cell lines [6, 8, 9, 22], ZL55 cells included [12] and that cisplatin activates ERK1/2 in ZL55 cells [15]. We showed that whereas Ptac2S activated all three MAPKs in ZL34 cells, in ZL55 cells it activated p38MAPK and JNK1/2 but not ERK1/2 (Fig 4A and ref. [12]). Pre-incubation with JNK1/2 inhibitor SP600125, or with the p38MAPK inhibitor SB203580, significantly reduced Ptac2S-induced cytotoxicity in both cell lines (Fig 4). Furthermore, SP600125 markedly inhibited Ptac2S-induced activation of p53 (Fig 4), thus suggesting that JNK1/2 mediates p53 induction. PD98059, an inhibitor of MEK1/2, the ERK1/2 upstream kinase, significantly increased Ptac2S-induced cytotoxicity, in ZL34 cells (Fig 4). Jointly these results indicated that JNK1/2 and p38MAPK are pro-apoptotic pathways whereas ERK1/2, in ZL34 cells, behaves like an anti-apoptotic survival pathway.

### Role of PKCs in Ptac2S-induced apoptosis in MPM cells

As the cellular effects of Ptac2S go together with the activation of various PKC isoforms, we here have studied their activation. In the previous study [12] in ZL55 cells, we showed that PKC-ε and PKC-δ translocated from the cytosol to the membranes; similarly to what happened with cisplatin [15], the cells treated with Ptac2S also show the proteolytic activation of PKC-δ. While the full-length PKC-δ moved to the membrane and nuclei, its fragment was located to the mitochondria. In contrast to cisplatin, the PKC-α was not activated (data not shown).

**Fig 5:**
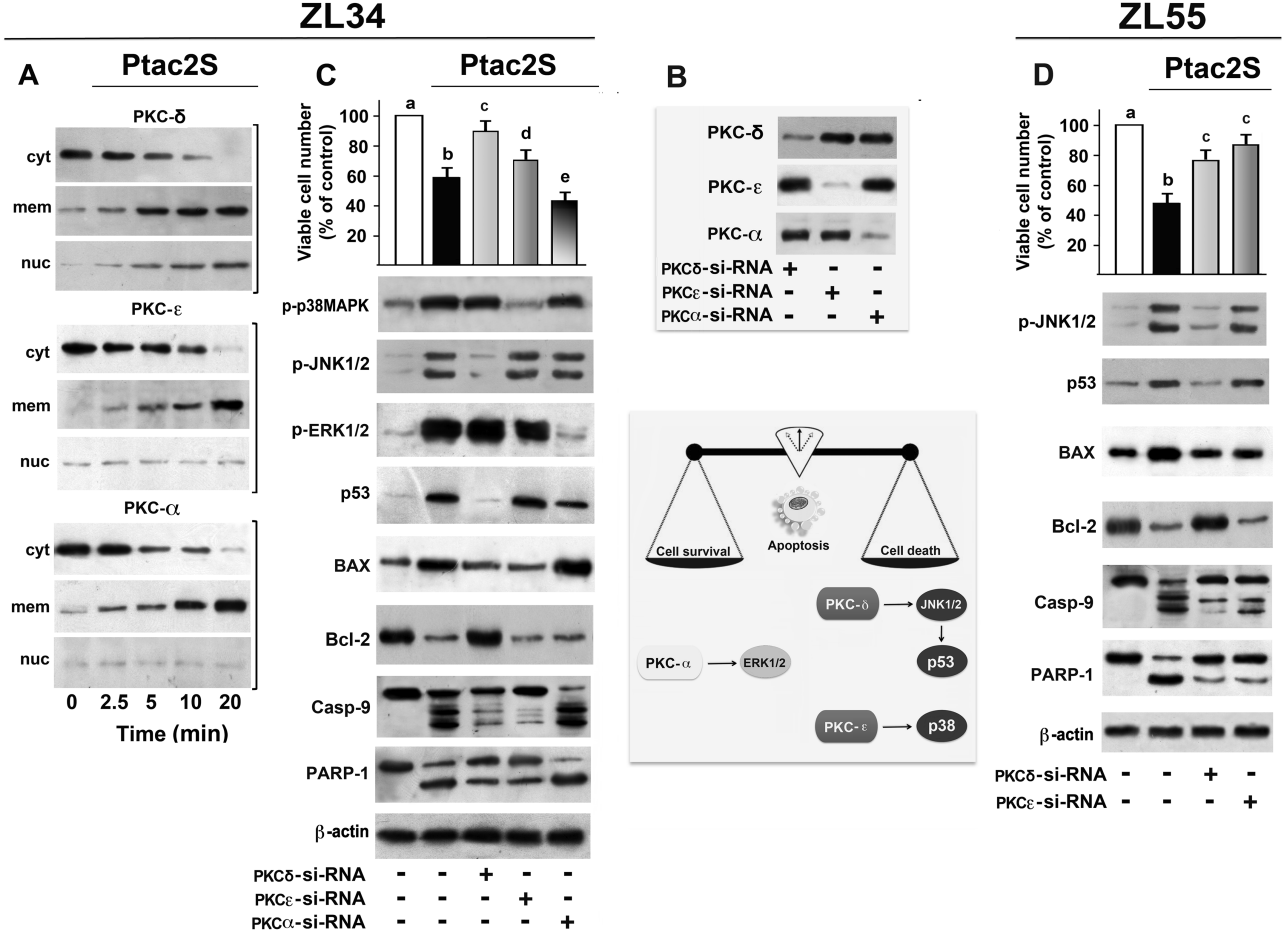
Role of PKCs in Ptac2S-induced apoptosis in MPM cells. **(A)** ZL34 cells were treated without or with 5 μM Ptac2S for the indicated times. For PKCs translocation studies, cytosol (cyt), membrane (mem), nuclei (nuc) fractions were analysed by western blotting with specific antibodies. The figures are representative of six independent experiments. (**B**) MPM cells were transfected with siRNA–PKC-δ or siRNA–PKC-ε or siRNA–PKC-α and then were incubated with 5 μM Ptac2S; western blotting of total lysates was then performed with specific anti-PKCs antibodies in order to show the decrement of PKC-δ, PKC-ε or PKC-α expressions. The figures are representative of six independent experiments. (**C**) ZL34 cells were transfected with siRNA–PKC-δ or siRNA–PKC-ε or siRNA–PKC-α whilst (**D**) ZL55 cells were transfected with siRNA–PKC-δ or siRNA–PKC-ε only and then incubated with Ptac2S. Viable cell number was determined 24 h later by SRB assay. The data are means ± S.D. of five different experiments run in eight replicates and are presented as percent of control. P < 0.0001 by one-way ANOVA (n = 5); values with shared letters are not significantly different according to Bonferroni/Dunn post hoc tests. Cytosolic or nuclear (for PARP-1) fractions were analysed by western blotting with antibodies against PKC-δ, PKC-ε, PKC-α phosphorylated p38MAPK, phosphorylated JNK1/2, phosphorylated ERK1/2, p53, BAX, Bcl2, caspase-9 (Casp-9) and PARP-1; ß-actin was used as a control for protein loading. Representative immunoblots of five experiments are depicted. **Inset**: crosstalk between MAPKs, p53 and PKCs pathways, key factors affecting cell death and survival in Ptac2S-treated MPM cells.

Here, the cytosol-to-membrane translocation of PKCs was followed by immunoblotting in ZL34 cells incubated with Ptac2S (0–20 min). Of the various isoforms expressed, PKC-ε, PKC-δ and PKC-α were activated by translocation: PKC-ε translocated to the plasma membrane and PKC-δ to plasma membrane and nucleus (Fig 5A). The roles of PKCs were evaluated using siRNA technique to inhibit PKC-ε, PKC-δ or PKC-α. After seeing by western blotting that PKC-siRNAs decreased PKC-ε, PKC-δ and PKC-α expressions (Fig 5B) it was shown that PKC-δ inhibition increased survival (Fig 5C and 5D) and decreased caspase-9 activation and PARP cleavage in ZL34 cells treated with Ptac2S (Fig 5C). Thus, in ZL34 cells the role of activated PKC-δ appears the same as the role it plays when the ZL55 cells are incubated with cisplatin [15] or with Ptac2S [12]. In addition, PKC-δ–siRNA (10 nM) inhibited Ptac2S-induced JNK1/2 phosphorylation, p53 and BAX induction as well as Bcl-2 down-regulation (Fig 5C and 5D), in both cell lines. PKC-ε–siRNA (10 nM) inhibited p38MAPK phosphorylation, caspase-9 activation and PARP cleavage but also increased the survival of Ptac2S-treated ZL34 cells (Fig 5C). PKC-α-siRNA (10 nM) inhibited the phosphorylation of ERK1/2 (Fig 5C) indicating that PKC-α activation is crucial for the survival of Ptac2S-treated ZL34 cells. The same results were obtained when 1 μM Go6976 (inhibitor of conventional PKCs) was used (data not shown).

## Discussion

MPM originates from mesothelium cells that form a specialised monolayer that line serous cavities of pleura, pericardium or peritoneum [23]. MPM cells can be either epithelioid or sarcomatoid [24] and several studies demonstrated a responsiveness to chemotherapeutics dependent from the phenotypes [25–26]. Therapies for MPM associates pemetrexed to cisplatin getting so a 40% response rate, and 3 months and 1 year average survival and median survival times, respectively [3, 27, 28]; the same happens associating carboplatin, liposomized doxorubicin and gemcitabine [29]. There is an urgent need for effective therapy since about 50% of MPM patients are primarily resistant and all eventually evolve resistance [30]. Ptac2S is a platinum complex holding 2 acetylacetonate ligands and dimethyl sulphide in the Pt coordination sphere with compelling biological activities [6–14, 21]. Unlike cisplatin, that has genomic (formation of DNA adducts) and non-genomic activities [6, 7], Ptac2S reacts poorly with nucleobases and has characteristic reactivity with sulphur ligands, indicating that cell targets could be protein aminoacid residues. This can make it inherently less capable of evoking chemo resistance [6]. In previous studies comparing Ptac2S and cisplatin activities, Ptac2S has shown a great anti-tumour activity *in vivo*, and a reduced nephrotoxicity and acute toxicity [11]; these results urged the present preclinical study in order to evaluate the therapeutic potential of Ptac2S also in mesothelioma. In addition, we showed that Ptac2S was more efficacious than cisplatin in inducing apoptosis in epithelioid ZL55 cells and, in a preclinical model based on injection of ZL55 cells, Ptac2S shows up an anticancer activity higher than that of cisplatin [12]. To investigate the difference in sensitivity between epithelioid and sarcomatoid mesothelioma cell lines to treatment with Ptac2S, we prepared xenograft models of sarcomatoid mesothelioma by injection of ZL34 cells. In Ptac2S group mice displayed an important curtailment of tumour volume at each time point compared with both not treated and cisplatin-treated mice (Fig 1). The *in vivo* effects of Ptac2S (53% reduction of tumour mass) were considerably greater than those of cisplatin (12% reduction).

*In vitro* antitumor activity was consistent with the *in vivo* sensitivity, since the cytotoxic effects of Ptac2S were grater than those elicited by cisplatin (Fig 2). In our experiments, the cleavage of PARP happens very quickly (though, already after just 3 hours in ZL55 cells, Ref. [12]) suggesting that Ptac2S causes rapid apoptosis onset. The caspase-7 cleavage pattern was detected earlier in cells treated with Ptac2S compared to cisplatin, and is similar to PARP proteolysis time course, hence supporting the study that shows that the proteolysis of PARP is due to caspase-7 [31]. Consistently with previous results, in Ptac2S-treated MPM cells the activation of caspase-9 occurred along with the activation of caspase-7, denoting the implication of the intrinsic pathway. Furthermore, after few hours of Ptac2S treatment Bcl-2 and BAX expression levels decreased and increased, respectively. In epithelioid ZL55 cells Ptac2S was approximately 12-fold more cytotoxic than cisplatin (IC_50_ were 0.98±0.14 μM for Ptac2S and 11.26±0.41 μM for cisplatin, see Ref [12]); such cell type was also significantly more sensitive to both cisplatin and Ptac2S than sarcomatoid ZL34 cells. *In vivo* we also noted a phenotype-dependent sensitivity to Ptac2S (ZL55 tumour size decreased to 38% [12], whilst ZL34 tumour size decreased to 47%), a result in agreement with the conclusion that patients with sarcomatoid tumours have a poor prognosis [23]. This result points out the necessity of a greater understanding of the relationship between MPM phenotype and the sensitivity of the cancerous cells such as the correlation to predictive markers, in order to increase clinical Pt derivatives efficacy. However, in both ZL34 and ZL55, Ptac2S induced apoptosis by up-regulating p53 protein and mRNA levels (Fig 3). Many pathways mediate the apoptosis due to p53, and among these there is one mediated by BAX proteins and by the pro apoptotic components of Bcl-2 protein family [32, 33]. BAX gene promoter contains several consensus sequences for p53 binding and is heavily trans activated by p53 [34]. BAX is able to foster cytochrome c release in the cytosol, thus activating caspase 9, which leads to apoptosis [35, 36]. In addition, BAX may bind to Bcl-2 inhibiting its apoptosis suppression function. P53 may suppress Bcl-2 expression [37]. Such down-regulation, noted in various models of apoptosis, diminishes Bcl-2 ability to heterodimerise with BAX [38]. In contrast, in mesothelioma cell lines and MPM cancer specimens, the defensive character of Bcl-2 is fewer evident [39, 40]. Albeit in other MPM cell lines are found small levels of Bcl-2 mRNA /protein, in ZL34 MPM cells, treatment with Bcl-2 antisense oligonucleotides reduced apoptosis threshold [41]. However, the transcriptional activation of p53 target genes and *de novo* synthesis of their products are not obligatory for p53 to induce apoptosis in certain experimental models. In the presence of actinomycin D or cycloheximides, which block RNA synthesis, p53 mediated apoptosis still occurred [42]. In Ptac2S-treated ZL34 and ZL55 cells, p53 regulated proapoptotic BAX and antiapoptotic Bcl-2 proteins *via* transcriptional regulation, as demonstrated by p53 inhibition. As stated above, activated MAPKs are involved in apoptosis [43] also when apoptosis is due to Ptac2S, as shown in human neuroblastoma and breast cancer cells [8, 9]. We have show that Ptac2S causes activation of p38MAPK in ZL55 cells [12], and in the present paper we also show that Ptac2S causes activation of MAPKs and that p38MAPK and JNK1/2 have a pro-apoptotic role in both ZL34 and ZL55. The activation of PKC-ε in Ptac2S treated cells is accountable for the sustained p38MAPK phosphorylation. The role of p38MAPK is underscored by the fact that its inhibition by PKC-ε-siRNA significantly decreased Ptac2S-induced cytotoxicity (Fig 5). Similarly, active p38MAPK is necessary for apoptosis in leukaemia cells [44], in CdCl_2_-treated promonocytic cells [45] and in cortical neuronal cells treated with calyculin A [46]. The blockage of p53 activation due to JNK1/2 inhibition may suggest that the activation of p53 signalling happens downstream of JNK1/2 (Fig 5). This is in disagreement with precedent studies showing that JNK1/2 is downstream of p53 [47, 48]. On the other hand, various studies demonstrated that JNK1/2 might modulate p53 and its targets and can positively affect apoptosis [49, 50]. Activation and stabilization of p53 by JNK1/2 signalling has been reported in mouse fibroblast [51] and in human multiple myeloma cells [52]. PKC-δ is a key element in apoptosis, as reported in HeLa cells [53], in acinar cells of the salivary glands [54] and in ZL55 cells [12,15].

In this work, we show that JNK1/2 activation is mediated by PKC-δ, similarly to what observed during apoptosis in acinar cells of the salivary glands [54] since JNK1/2 inhibition revert the apoptotic effects of Ptac2S. With regard to PKC-α, it is able to protect from apoptosis [55]. PKC-α phosphorylates Bcl-2 *in vitro*, and over expression of PKC-α increases phosphorylation of Bcl-2 and suppresses apoptosis of pre-B REH cells [56]. Contrariwise, apoptosis of prostatic cancerous cells is provoked by PKC-α over expression or activation through phorbol-12-myristate-13-acetate [57].

In this report we show that PKC-α was activated by Ptac2S in ZL34 cells, starting an antiapoptotic program able to activate a signalling pathway comprehending ERK1/2 (ERK1/2 phosphorylation was blocked by siRNA-PKC-α, Fig 5). ERK1/2 phosphorylation may wield either an anti- [58] or a pro- apoptotic [59] result relying on cellular context and/or as yet unclear regulatory mechanisms. Coherent with a pro-survival action of ERK1/2 [60,61] we here supplied evidences that ERK1/2 is involved in drug resistance induction. Indeed, the cytotoxic effects of Ptac2S increased when the Ptac2S-provoked ERK1/2 phosphorylation was inhibited (Fig 5).

We conclude by pointing out that Ptac2S is effective in pleural mesothelioma and that these new acquisitions enhance the knowledge of the anti tumour activity of this compound. Finally, it appears facilitated the ability to translate this information into clinical practice, in order to improve the response to chemotherapy of resistant tumours.

## Abbreviations

FBS: fetal bovine serum
MPM: malignant pleural mesothelioma
MTT: 3-(4,5-dimethylthiazol-2-yl)-2,5-diphenol tetrazolium bromide
PK: pharmacokinetics
Ptac2S: [Pt(*O*,*O′*-acac)(γ-acac)(DMS)]
SRB: sulforhodamine B
