# Correction: Apoptosis by [Pt(*O*,*O′*-acac)(γ-acac)(DMS)] requires PKC-δ mediated p53 activation in malignant pleural mesothelioma

**DOI:** 10.1371/journal.pone.0193030

**Published:** 2018-02-12

**Authors:** Antonella Muscella, Carla Vetrugno, Luca Giulio Cossa, Giovanna Antonaci, Amilcare Barca, Sandra Angelica De Pascali, Francesco Paolo Fanizzi, Santo Marsigliante

There are errors in the Funding section. The correct funding information is as follows: This work was supported by the Ministry of Education, University and Research (FIRB CAROMICS RBAP11B2SX_008) and by the Interuniversity Consortium of Chemistry Research in Metals in Biological Systems (CIRCMSB), Bari, Italy.
